# Comparative efficacy of intravitreal pharmacotherapy for macular edema secondary to retinal vein occlusion: A protocol for the systematic review and network meta-analysis

**DOI:** 10.1097/MD.0000000000022267

**Published:** 2020-09-18

**Authors:** Yun Zhang, Jianan Duan, Tiancong Chang, Xun Li, Miao Wang, Meixia Zhang

**Affiliations:** a Department of Ophthalmology; b National Clinical Research Center for Geriatrics, West China Hospital, Sichuan University, Chengdu, Sichuan, China.

**Keywords:** intravitreal pharmacotherapy, macular edema, network meta-analysis, retinal vein occlusion

## Abstract

**Background:**

Multiple intravitreal pharmacotherapies including different anti-vascular endothelial growth factors (VEGF), intravitreal steroids, and combined therapy with anti-VEGF and steroids are available for patients with macular edema secondary to retinal vein occlusion (RVO). However, the recommendation of multiple therapies remains unknown. This study aims to evaluate the efficacy and safety of multiple intravitreal pharmacotherapies in patients with macular edema secondary to RVO.

**Methods:**

We will systematically search the PubMed, Embase, and the Cochrane library for eligible studies. Randomized controlled trials (RCTs) with intravitreal pharmacotherapies for patients with macular edema secondary to RVO will be included. The Cochrane Collaboration's tool will be used to assess the risk of bias in the randomized trial. The primary outcome is the mean change in BCVA from baseline. The secondary outcomes are the proportion of patients who gained ≥15 letters in BCVA from baseline, the mean change in central retinal thickness from baseline and the number of serious adverse events.

**Results:**

The result will obtain a comprehensive treatment recommendation for macular edema secondary to RVO.

**Conclusion:**

The results of the network meta-analysis will be submitted in a peer-reviewed journal for publication.

**Ethical statement:**

This article does not contain any studies with human or animal subjects performed by any of the authors.

## 1 Introduction

Retinal vein occlusion (RVO) is a common retinal vascular disease, injuring visual acuity frequently. Currently, RVO is regarded as branch RVO (BRVO), central RVO (CRVO) and hemiretinal vein occlusion according to anatomic location of occlusion.^[1,2]^ Previously, epidemiologic studies showed that age- and sex-standardized prevalence was 5.20‰ for any RVO, 4.42‰ for BRVO, and 0.80‰ for CRVO.^[3]^ Tien T. Wong investigated that the present prevalence of RVO in Asia is 0.72%, which was estimated to be rising continually. Moreover, the amounts of RVO patients will reach up to 21 million by the year 2040.^[4]^ The risk factors of RVO occurrence include arterial hypertension, age, diabetes, and hypercoagulability.^[5]^ Variable clinical characteristics of RVO are reported. For example, unilateral painless vision loss is a common symptom, which may be abrupt and severe, or asymptomatic.^[6]^ The sharp decrease of VA may be as result of macular edema and retinal neovascularization secondary to RVO. In addition, extensive capillary nonperfusion could induce neovascular glaucoma.^[7,8]^ Over the past decades, the recommended treatments for RVO comprised laser photocoagulation, anti-vascular endothelial growth factor (anti-VEGF) intravitreal injections and intravitreal steroid injections. However, all current treatments characterize by achievement and futility and the consensus has not been reached on standard of care for macular edema secondary to RVO.

The earliest treatment option was laser photocoagulation.^[9]^ The Branch Vein Occlusion Study (BVOS) trial first established standards for treatment management of patients with BRVO.^[10]^ It also verified the improvement of visual acuity and lower risk of vitreous hemorrhage in the treatment of macular edema secondary to BRVO with the macular grid laser photocoagulation. But the Central Vein Occlusion Study (CVOS) failed to demonstrate the benefit from grid laser photocoagulation for macular edema secondary to CRVO, only an advantageous treatment trend in younger patients.^[11,12]^ Moreover, laser photocoagulation cannot perform in retinal swelling and hemorrhage due to laser energy absorbed and reduced. Therefore, laser photocoagulation might lead to limited visual improvement and generally only used as a rescue therapy for macular edema secondary to RVO.

Corticosteroids have been proven to reduce retinal vascular permeability and inflammation and associated with the regulation of VEGF-A expression.^[13]^ Therefore, intravitreal steroids became another option for RVO patients. But the SCORE study showed that the long-term safety and efficacy of intravitreal triamcinolone acetonide (TA) is not much beneficial for macular edema related to BRVO.^[14]^ The SCORE study for CRVO indicates that intravitreal TA may produce visual functional and anatomical improvement of macular edema secondary to CRVO but the effects are of short duration, with more complications related to the steroid, increasing intraocular pressure and cataract et al.^[15]^ Recently, intravitreal dexamethasone implant (Ozurdex, Allergan, Inc, Irvine, CA), providing sustained release of the potent corticosteroid, approved for the treatment of macular edema related to RVO in many countries. The GENEVA study demonstrated there were significant visual acuity improvement and anatomic recovery at 90 days that were lost at 180 days for RVO patients.^[16]^ Overall, the efficacy of corticosteroids for macular edema due to RVO may be superior to the natural history of the disease, but possible side effects cannot be ignored.

In recent years, repeated intravitreal injections of anti-VEGF has been the standard care for treating RVO, including on-label use of ranibizumab (Lucentis; Genentech, Inc., South San Francisco, CA) and aflibercept (Eylea, Regeneron, Tarrytown, NY, and Bayer HealthCare, Berlin, Germany), off-label use of bevacizumab (Avastin; Genentech, Inc, South San Francisco, CA) and conbercept (KH902, Chengdu Kanghong Biotech Co, Ltd, Sichuan, China). RVO causes hypoxia in the retinal system and elevates the level of VEGF, which is related to the clinical severity.^[17]^ Therefore, anti-VEGF injection is applied as first-line therapy for RVO, including BRVO and CRVO.^[18]^ Multiple studies have demonstrated the efficacy of anti-VEGF agents both visual acuity improvement and macular edema reduction.^[19–24]^ However, this efficacy potentially needs multiple intravitreal injection which increase the risk of hemorrhage, retinal ischemia, and vitreous traction, et al.^[25]^ Besides, some studies did combination therapy with TA and anti-VEGF, anti-VEGF combined with laser photocoagulation which tried to reduce the injection number and maximize visual acuity outcome and anatomical structure.^[26,27]^ The further investigations are still needed for efficacy and safety.

However, the most effective treatment for macular edema secondary to RVO has not yet been confirmed because of the shortage of head-to-head randomized controlled trials (RCTs) and the limitations of traditional meta-analyses. Several traditional meta-analyses were performed on the therapies of RVO, but they are neither including all the comparisons among laser, steroids nor anti-VEGF injections with dose and therapeutic regimen distinguished. Network meta-analysis includes both direct and indirect comparison and overcomes the limitation of tradition meta-analysis. Its advantages lie in improving the accuracy of evaluation for the current treatments for RVO. Therefore, we plan to perform a systematic review and network meta-analysis to evaluate the safety and efficacy of current therapy for RVO, including laser photocoagulation, steroids, anti-VEGF injections, and combination therapies.

## 2 Methods

The protocol adheres to preferred reporting items for systematic review and meta-analysis protocol (PRISMA-P) checklist.^[28]^ The network meta-analysis will be conducted following PRISMA for Network Meta-Analyses guidelines.^[29]^ The study has been registered in INPLASY (INPLASY202070012). The study does not involve human subjects and does not need ethical approval and patient consent.

### 2.1 Eligibility criteria

The PICOS strategy (patients, intervention, comparisons, outcome, and study design type) determines the eligibility criteria for the study.

#### 2.1.1 Patients and comparison of interventions

We will include studies focusing on patients with macular edema secondary to RVO, including BRVO and CRVO, respectively. Comparisons of laser photocoagulation, intravitreal steroid, different anti-VEGF monotherapies, and combined therapy with laser photocoagulation and anti-VEGF will be included. No further restrictions will be made on age, ethnic distribution, and gender.

#### 2.1.2 Outcomes

The primary outcome is the mean change in BCVA from baseline. The secondary outcomes of the study include the proportion of patients who gained ≥15 letters in BCVA from baseline, the mean change in central retinal thickness from baseline and the number of serious adverse events. All the outcomes will be measured and analyzed separately in BRVO and CRVO.

#### 2.1.3 Study design

Study designs of interest will include only published RCTs comparing laser photocoagulation or intravitreal steroid or different anti-VEGF monotherapies or combined therapy with laser photocoagulation and anti-VEGF for the treatment of macular edema related to BRVO or CRVO. There is no language restriction.

### 2.2 Information sources and search strategy

We will search the following databases: PubMed, Embase, and the Cochrane library. All databases will be systematically searched from implementation to January 1, 2020. We also will identify additional relevant studies in the reference catalog.

Search strategy of PubMed was as follows:

**#1** (((((retinal vein occlusion) OR “Retinal Vein Thromboses”) OR “Retinal Vein Thrombosis”) OR “Retinal Vein Occlusions”))
**#2** ((((macular edema) OR “Cystoid Macular Edema”)) OR “macular oedema”)
**#3** #1 AND #2
**#4** ((((((((“Randomized Controlled Trial” [Publication Type]) OR “Controlled Clinical Trial” [Publication Type]) OR “randomized” [tiab]) OR “placebo” [tiab]) OR “Clinical Trials as Topic”[Mesh:NoExp]) OR “randomly” [tiab]) OR “trial” [ti])) NOT ((“Animals” [mh]) NOT “ humans” [mh])
**#5** #3 AND #4

### 2.3 Selection process and data management

Two independent reviewers will complete study selection and data management. Reviewers will evaluate the study titles and abstracts to include articles that meet the inclusion criteria. Disagreements will be resolved by discussion.

For studies that meet the eligibility criteria, we will extract data from articles into a standardized form. The data includes characteristics of the study, data needed for quality assessment, baseline characteristics of participants (mean age, sample size, types of interventions received, drug dosages, therapeutic regimens) and outcomes indicators.

If standard deviations or standard errors were not reported for continuous outcomes, we will first calculate effect size in Review Manager version 5.3 based on the reported data such as 95% confidence intervals (CIs) or *P*-value. If the effect size cannot be calculated, we will contact the authors to obtain additional data. If there is no response, we will send two email reminders to study authors.

### 2.4 Risk of bias of individual studies

The risk of bias in individual studies will be assessed by two independent researchers following Cochrane Collaboration's tool for assessing the risk of bias in randomized trials.^[30]^ Assessment of the risk of bias will be based on the following domains: sequence generation, allocation concealment, blinding of participants and researchers, incomplete outcome data, selective reporting, and other bias random.^[30]^ Disagreements will be resolved through discussion.

### 2.5 Data synthesis and statistical analysis

#### 2.5.1 Measures of treatment effects

We will calculate continuous data using the weighted mean difference (WMD) with 95% CIs and dichotomous variables using relative risk (RR) with 95% CIs. The two-sided *P* < .05 can be assumed if 95% CIs do not include 0 at conventional levels of statistical significance.^[31]^ We will assess heterogeneity using the chi-square test and the *I*^2^ statistic. Chi-square test with the significance set *P* < .1 or *I*^2^ > 50% indicates statistical heterogeneity.^[32]^

#### 2.5.2 Data analysis

First, we will perform traditional pairwise meta-analyses with a random-effects model for every head-to-head comparison involving at least two RCTs.^[33]^ Then, we will conduct a network meta-analysis with a Bayesian random-effects model. Apart from pooled WMDs or RR with 95% CIs, relative ranking probability of different therapies for macular edema due to RVO will be presented through surface under cumulative ranking curve (SUCRA) values. SUCRA values will be expressed as percentages of efficacy or safety of each intervention, and a larger the SUCRA value indicates the better the rank.^[34]^ For each analysis, we will assess the inconsistencies between direct and indirect evidence in the network using the node splitting method.^[35]^ Comparison-adjusted funnel plots will be drawn to estimate publication bias in the network meta-analysis.^[36]^ We will draw network plots to present the geometry of the network meta-analyses.

All analyses involved will be performed using R v3.5.0 (gemtc package and rjags package), and Stata version 14 (Stata Corp, College Station, TX).

## 3 Discussion

Currently, the optimal therapy for macular edema secondary to RVO is still uncertain. Laser photocoagulation, intravitreal steroid, different anti-VEGF monotherapies, and combined therapy with laser photocoagulation and anti-VEGF are all available options for patients with macular edema related to BRVO or CRVO. To the best of our knowledge, this study will be the first network meta-analysis to simultaneously compare multiple intravitreal pharmacotherapies for macular edema related to RVO. We expect our study can get a ranking of different therapies and give recommendations for ophthalmologists.

## Author contributions

**Conceptualization:** Yun Zhang, Meixia Zhang.

**Data curation:** Yun Zhang.

**Investigation:** Jianan Duan, Tiancong Chang.

**Methodology:** Yun Zhang, Xun Li, Miao Wang.

**Writing – original draft:** Yun Zhang.

**Writing – review & editing:** Jianan Duan, Meixia Zhang.

## Abbreviations

Abbreviations: BCVA = best-corrected visual acuity, BRVO = branch retinal vein occlusion, CRVO = central retinal vein occlusion, RVO = retinal vein occlusion, TA = triamcinolone acetonide, VEGF = vascular endothelial growth factor.
